# Knowledge, attitude and practice of antibiotic use among university students: a cross sectional study in UAE

**DOI:** 10.1186/s12889-019-6878-y

**Published:** 2019-05-06

**Authors:** Ammar Jairoun, Nageeb Hassan, Abdelazim Ali, Obaida Jairoun, Moyad Shahwan

**Affiliations:** 1 College of Pharmacy and Health Sciences, Ajman University, Ajman, UAE; 2 College of Dentistry, Ajman University, Ajman, UAE

**Keywords:** Antibiotic usage, Knowledge, Attitude, Practice, KAP, Medical students

## Abstract

**Background:**

Antibiotic resistance became a marker of irrational and overuse of these medicines in many countries. This study aims to evaluate the knowledge, attitude and practice (KAP) of medical students (MS) and non-medical students (NS) towards antibiotic use in the United Arabs Emirates (UAE).

**Method:**

A descriptive cross-sectional study was conducted amongst 1200 MS and NS from Ajman University in UAE. A self-administered questionnaire was used to assess the knowledge, attitude and practice of antibiotic use. The later was composed into knowledge, attitude and practice of antibiotic use. Descriptive analysis was used to analyse the qualitative variables while quantitative variables were summarised using mean ± Standard Deviation (±SD). A Chi-square test was used to compare differences in the proportions of qualitative variables. Unpaired student’s t-test was used to test the average differences in quantitative variables across medical and non-medical students. A *p* < 0.05 was considered statistically significant.

**Results:**

One thousand two hundred students (MS: 600 and NS: 600) were considered valid for analysis. On average, participants scored higher in attitude score followed by knowledge and practice scores. The average attitude score was 76% (95% CI: [75, 78%]) compared to 59% for knowledge (95% CI: [58, 60%]) and 45% (95% CI: [44, 47%]) for practice. The results suggest that overall, medical students scored remarkably better than non-medical students on KAP of antibiotic use, respectively (*p* = 0.0001), (p = 0.000) and (*p* = 0.002).

**Conclusion:**

The students’ knowledge, attitude and practice regarding antibiotic use, which drive the practice of self-medication, reflect a gap in medical curricula in UAE institutes and medical colleges.

## Background

Antibiotics were called “magic bullets” for quite some time; however, these magic bullets were not always magical enough to survive some serious downsides. The use and misuse of antibiotics induce selection pressure, resulting in the development of resistance traits in bacterial populations [1]. However, the problem was not the antibiotics themselves as they remained one of the most effective weapons against diseases; in fact, the problem lied in the drugs’ use. Overusing antibiotics or using them irrationally can easily result not only in the emergence of resistant bacterial strains but also in adverse reactions and can also result in an economical burden on the national health system [2]. The World Health Organization set the theme of the World Health Day as Combat Antimicrobial Resistance: No Action Today, No Cure Tomorrow’ [3]. According to a September 2013 report from the U.S. Center for Disease Control and Prevention (CDC), treatment of antibiotic-resistant infections adds $35 billion to health care costs and 8 million hospital days per year in the United States [4]. Antibiotic use might be influenced by several factors such as unregulated drug availability, relaxed health policies concerning regulations on antibiotic use, procurement of antibiotics without prescription (over-the-counter acquisition), patients’ knowledge and attitudes towards antibiotic use, self-medication, physicians’ knowledge and experiences and patient-prescriber interaction. Therefore, controlling antibiotic use requires feasible means of intervention. Many strategies have been proposed for the use of antibiotics such as a formulary replacement or restriction, health care provider education, feedback activities, approval requirement from an infectious disease specialist for the drug prescription and a more rational use of antimicrobial agents all over the world [5, 6]. The students can play a crucial role in reducing the inappropriate use of antibiotics; in this context, an increasing number of research reports in the literature have focused their attention on investigating the KAP of medical and non-medical students towards antibiotics use. A survey of 2500 medical and non-medical students in China indicated that medical students performed better than non-medical students in terms of knowledge and attitude towards antibiotic use [7]. This is similar to the findings of another study conducted in Chennai among medical and paramedical students [8]. In a study in Malaysia by Ahmad et al. [9] with third- and fourth-year pharmacy students, sufficient knowledge but poor attitude of pharmacy students regarding antibiotics use was reported. Other studies in India [10, 11] reported poor knowledge regarding the antibiotic spectra, indications, side effect and their correct use. Moreover, students’ knowledge regarding antibiotic resistance and antibiotic prescribing was moderate as well as the attitude toward the cause of resistance [12]. Another study [13] revealed good understanding and awareness regarding antibiotic resistance; this was consistent with the study of Khan and Banu [14] which revealed that majority of the students were aware of antimicrobial resistance and its consequences. Another cross-sectional study conducted among university undergraduates to assess their knowledge and practice towards antibiotic use and reported poor knowledge of the right sources of antibiotic; furthermore, majority of them used the same antibiotics as previously prescribed by their doctors to treat their perceived infections while 51.2% kept leftover antibiotics for future use [15]. In Italy, a study performed in Torino University among health care profession students also revealed the existing gap between knowledge and practice [16].

### Aim of the study

To our knowledge, no study was conducted in Arab countries and Gulf Cooperation Council (GCC) countries, particularly in United Arab Emirates (UAE) evaluating the Knowledge, attitude and practice (KAP) of medical students towards antibiotic use. Therefore, this study aims to investigate medical students’ KAP in relation to antibiotic use by controlling with non-medical students.

## Methods

A cross-sectional survey carried out among randomly selected MS and NS from Ajman University in the UAE from January to May 2015. The targeted respondents for the survey were undergraduate students from first to fifth years in medical colleges and from first to fifth year in non-medical colleges. In order to overcome the multiplicity of sources of data collection that might confuse the assessment of study results, the directory of Admission and Registration Department of Ajman University was used as the sampling frame. This directory was considered a pre-existing frame with officially recognized and listed information on students’ names, university ID numbers, major, age, gender, nationality and contact numbers that were updated regularly. From the Excel sheet of the sampling frame, 600 MS from faculties of medical colleges and a paired 600 NS from faculties of non-medical colleges were enrolled in this study. The method of “Simple random sample selection” was followed. In this method, individuals were selected randomly and not more than once to avoid bias that could negatively affect the result’s validity. A self-administered questionnaire was distributed randomly to the participants during lectures (at the end of their classes) and a signed consent form was obtained from the participants.

After referring to previous similar studies [7, 11]. regarding KAP of antibiotic use in the literature, a structured self-administered was designed and adapted to cover all the main key points of the research and in a way that suit with local population of UAE.

The questionnaire was then reviewed and assessed by subject experts for its content, design, relevance, readability and comprehension. The questionnaire was validated by seven lecturers from the discipline of clinical pharmacy at Ajman university and it was examined for content relevance and appropriateness. Minor modifications were done based on their comments. Moreover, quantitative content validity of the instrument was ascertained based on Lawshe’s Content Validity [17]. A content validity ration (CVR) was calculated for each item and all items reported 0.71 CVR score. Items with CVR score of 0.7 or above were selected acceptable and if the item does not reach this threshold, it would normally be deleted from the final instrument [18]. Then, content validity index (CVI) was obtained by calculating the mean of the CVR values for all items meeting the CVR threshold of 0.70 and retained for the final instrument. The final reported CVI value for the instruments was 0.71 which indicating acceptable content validity of the entire instrument [19]. In addition, for reliability, a pilot study was done on 100 students from Ajman university and necessary changes were made accordingly. The participants who were participated in pilot study have been excluded from final analysis. The reliability analysis of the instrument was performed by calculating Cronbach’s α value. The Cronbach’s α value of the questionnaire was 0.73, indicating acceptable internal consistency.

The first section covered participants’ demographic data such as major and grade of education. The second section covered knowledge of antibiotic use and comprised 12 questions. The third section assessed the attitude of respondents regarding antibiotic use and had six questions. The fourth section explored the self-medication practices of students and had 15 questions.

All questions were coded and then imported to SPSS version 23 for analysis. Descriptive analysis was used to analyse the qualitative variables while quantitative variables were summarized using mean ± Standard Deviation (±SD). Graphical representations were provided for all relevant variables. The Chi-square test was used to compare differences in the proportions of qualitative variables. Unpaired student’s t-tests were used to test the average differences in quantitative variables across medical and non-medical students. A *p* < 0.05 was considered statistically significant. Three scores were created to measure the knowledge, attitude and practice of antibiotic use. Each score was defined as the proportion of questions for which the answers were correct. These three scores range from 0 to 100% and might be used as good approximation of the overall KAP. Shapiro wilk test was carried out to test the normality of the knowledge, attitude and practice scores. The results showed that there were no statistically significant deferens from the normal distribution for the knowledge score (*P* = 0. 87), attitude score (P = 0. 077), and practice score (P = 0. 063).

## Results

### Characteristics of the study population

A total of 1200 subjects participated in the study and completed the entire questionnaire. Among these participants, 600 (50%) were medical students constituting the primary target group and 600 (50%) were non-medical students mainly used as control. Fifty-five percent (55.5%) were first-year students and 44.5% were final-year students.

### Knowledge on antibiotic use: analysis of overall knowledge score

**Fig. 1:**
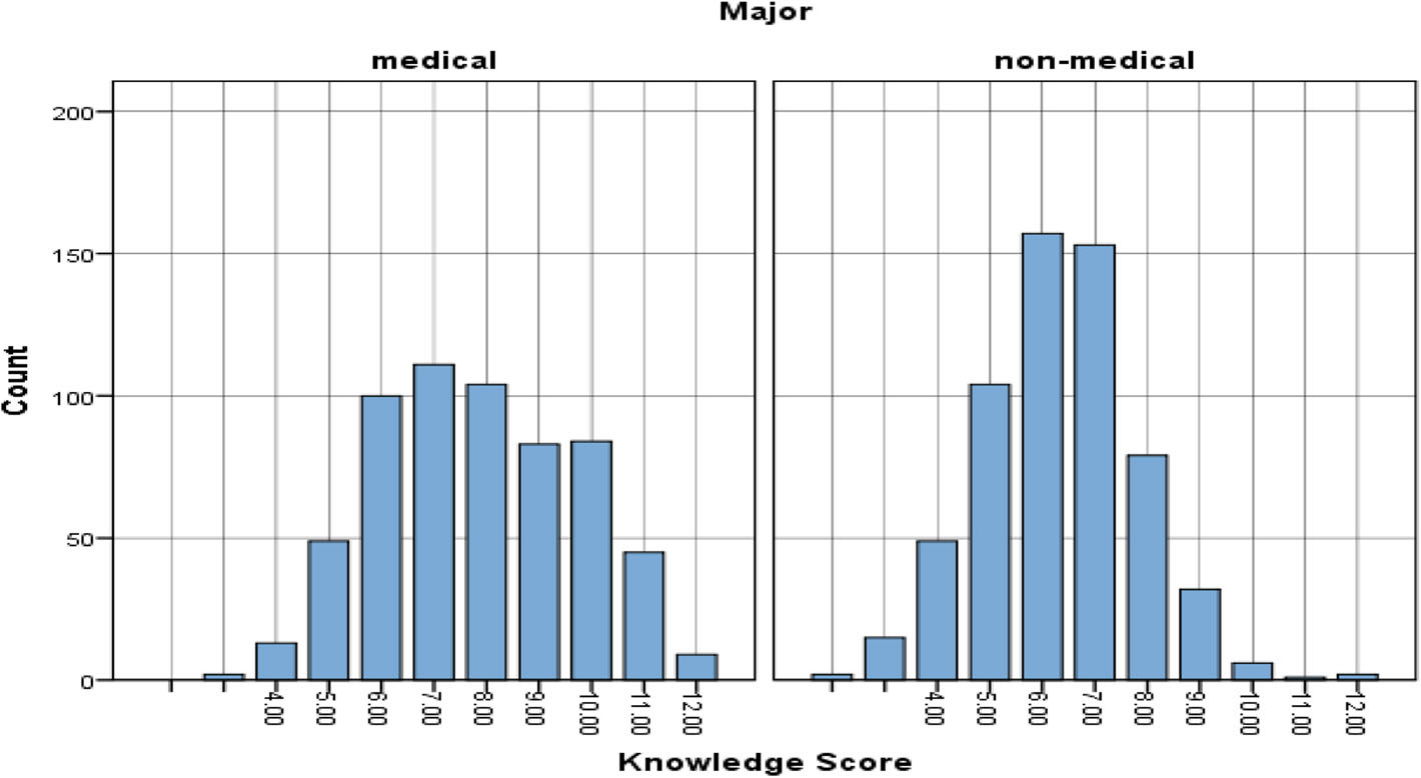
Histogram of knowledge score by major. Figure 1 shows the distribution of overall knowledge scores among medical and non medial students. The values in the x axis represents the total number of correct answers. Y axis represents the count

The average knowledge score was 59% with a 95% confidence interval (CI) [58, 60%] This means that on a knowledge scale of 0 to 100, participants scored an average of 59 points in the knowledge of antibiotic use. Table 1 shows the distribution of knowledge scores according to the major. MS scored relatively higher than NS in knowledge of antibiotic use (*p* = 0.000). Their average score was 65% compared to 53% of NS. The same pattern of results was observed between first-year MS and NS (p = 0.000) and final-year MS and NS (p = 0.000) (see Table 1 and Fig. 1). The results of each of the questions related to the knowledge of antibiotic usage among MS and NS were analysed using χ2 test (see Table 2).

**Table 1:** Medical and non-medical students average score on knowledge, attitude and practice of antibiotic use

Student Score	Overall average 95% CI	Whole average 95% CI			1st year average 95% CI			Final year average 95% CI		
		MS	NS	p	MS	NS	p	MS	NS	P
Knowledge	59 (58–60)	65 (64–67)	53 (52–54)	0.000	59 (57–60)	53 (51–54)	0.000	75 (74–77)	53 (51–54)	0.000
Attitude	76 (75–78)	80 (78–81)	73 (71–75)	0.000	77 (75–79)	72 (69–74)	0.001	84 (82–86)	75 (72–77)	0.000
Practice	45 (44–47)	47 (46–49)	43 (41–45)	0.002	47 (44–49)	45 (42–47)	0.232	49 (46–51)	42 (39–45)	0.001

*p < 0.05; #Significance; 95% CI confidence interval

**Table 2:** Number and percentage of the questions on the knowledge of antibiotic use

Question (correct response)	Total % (n/N)	Whole n (%)				1st year n (%)				Final year n (%)			
		MS	NS	χ2	p	MS	NS	χ2	p	MS	NS	χ2	p
Are there bacteria in human body which are good for our health? (Yes)	1169 (97.4%)	594 (99%)	575 (95.8%)	12	0.001	361 (98.6%)	281 (93.7%)	11.7	0.001	233 (99.6%)	294 (98%)	2.5	0.113
Can antibiotics be used to cure Infections caused by bacteria (Yes)	1097 (91.4%)	560 (93.3%)	537 (89.5%)	5.6	0.018	332 (90.7%)	260 (86.7%)	2.7	0.1	228 (97.4%)	277 (92.3%)	6.7	0.01
Can antibiotics be used to cure infections caused by viruses (No)	576 (48%)	395 (65.8%)	81 (30.2%)	153	0.000	215 (58.7%)	93 (31%)	51	0.000	180 (77%)	88 (29.3%)	119	0.000
Do you think the use of antibiotics will speed up the recovery of cold, cough and other diseases (No)	356 (29.7%)	205 (34.2%)	151 (25.2%)	11.6	0.001	112 (30.6%)	80 (26.7%)	1.2	0.256	93 (39.7%)	71 (23.7%)	16	0.000
Antibiotics are obtainable Without interference of a doctor at drug stores or pharmacies (No)	661 (55.1%)	318 (53%)	343 (57.2%)	2.1	0.147	213 (58.2%)	185 (61.7%)	0.83	0.364	105 (45%)	158 (52.7%)	3.2	0.074
Have you heard of resistance of bacteria (Yes)	914 (76.2%)	507 (84.5%)	407 (67.8%)	46	0.000	285 (77.9%)	194 (64.7%)	14.2	0.000	222 (95%)	213 (71%)	49.6	0.000
Do you think frequent use of antibiotics will decrease the treatment when using the antibiotic again (Yes)	913 (76.1%)	487 (81.2%)	426 (71%)	17	0.000	294 (80.3%)	203 (67.7%)	14	0.000	193 (82.5%)	223 (74.3%)	5.1	0.024
Is the efficacy better if the antibiotics are newer and the price is higher (No)	833 (69.4%)	435 (72.5%)	398 (66.3%)	5.4	0.020	258 (70.5%)	200 (66.7%)	1.1	0.289	177 (75.6%)	198 (66%)	5.8	0.016
Amoxicillin is antibiotic (Yes)	717 (59.8%)	414 (69%)	303 (50.5%)	42.7	0.000	193 (52.7%)	140 (46.7%)	2.4	0.119	221 (94.4%)	163 (54.3%)	104.7	0.000
Penicillin is antibiotic (Yes)	685 (57.1%)	381 (63.5%)	304 (50.7%)	20.2	0.000	193 (52.7%)	166 (55.3%)	0.449	0.503	188 (80.3%)	138 (46%)	65.2	0.000
Tetracycline is antibiotic (Yes)	353 (29.4%)	237 (39.5%)	116 (19.3%)	58.7	0.000	73 (19.9%)	63 (21%)	0.113	0.737	164 (70.1%)	53 (17.7%)	149.7	0.000
Definition of drug susceptibility testing of bacteria (Yes)	220 (18.3%)	169 (28.2%)	51 (8.5%)	77.5	0.000	53 (14.5%)	29 (9.7%)	3.5	0.060	116 (49.6%)	22 (7.3%)	122.4	0.000

*p < 0.05; #Significance; χ2 = chi square test.; n (%): Frequency (Percentage)

### Attitude on antibiotic use: analysis of overall attitude score

**Fig. 2:**
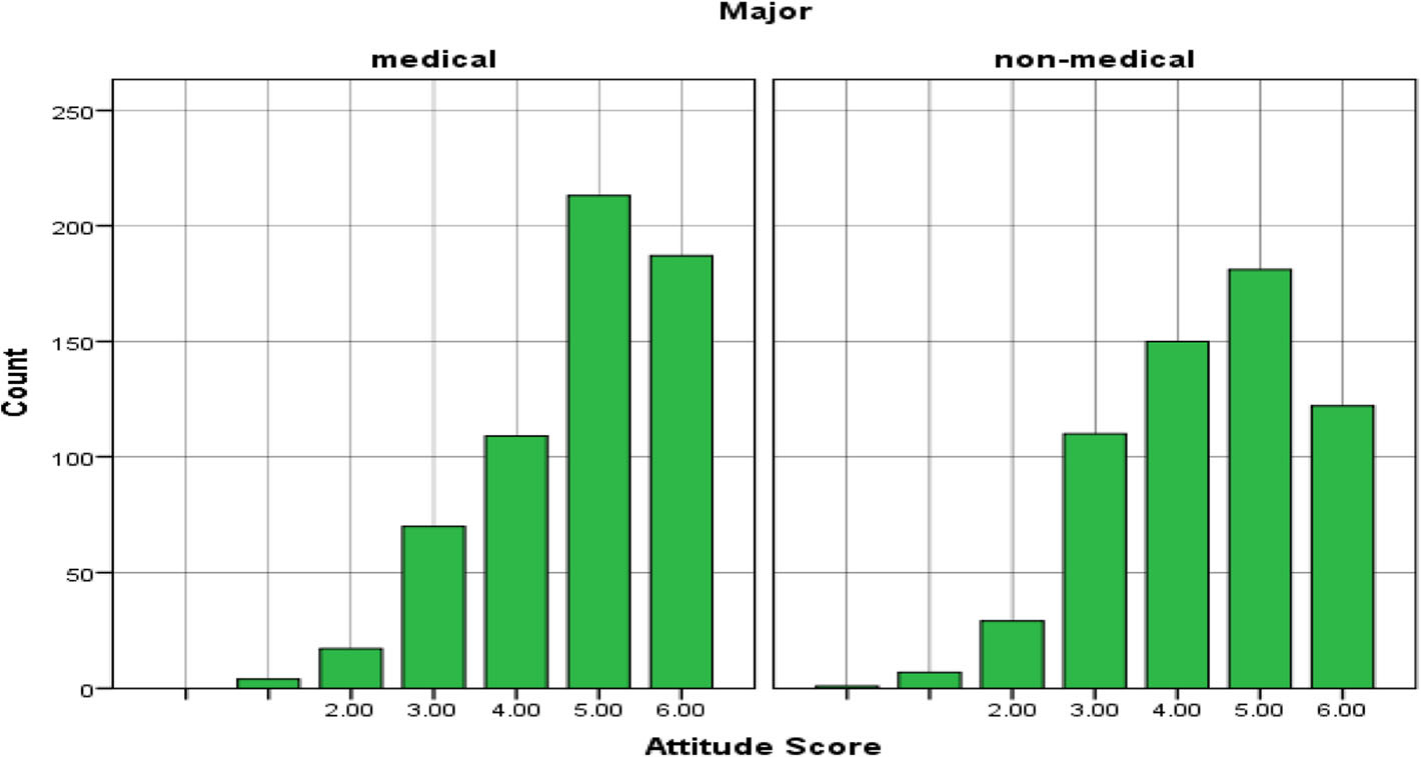
Histogram of attitude score by major. Figure 2 shows the distribution of overall attitude scores among medical and non medial students. The values in the x axis represents the total number of correct answers. Y axis represents the count

The average attitude core was 76% with a 95% confidence interval (CI) [75, 78%] This means that on an attitude scale of 0 to 100, participants scored an average of 76 points in the attitude towards antibiotic use. Table 2 shows the distribution of attitude score according to the major. MS scored relatively higher than NS in attitude towards antibiotic use (*p* = 0.000). Their average score was 80% compared to 73% in NS. The same pattern of results was observed between first year MS and NS (*p* = 0.001) and final MS and NS (p = 0.000) (see Table 1 and Fig. 2). The results of each of the questions related to the attitude towards antibiotic usage among medical and non-medical students were analysed using χ2 test (see Table 3).

**Table 3:** Number and percentage of the questions on the attitude of antibiotic use

Question (correct response)	Total % (n/N)	Whole n (%)				1st year n (%)				Final year n (%)			
		MS	NS	χ2	p	MS	NS	χ2	p	MS	NS	χ2	p
There is abuse on antibiotics at present. (Yes)	1063 (88.6%)	548 (91.3%)	515 (85.8%)	9	0.003	329 (89.9%)	256 (85.3%)	3.2	0.073	219 (93.6%)	259 (86.3%)	7.4	0.007
Antibiotic resistance a problem in UAE (Yes)	534 (44.5%)	287 (47.8%)	247 (41.2%)	5.4	0.020	152 (41.5%)	105 (35%)	3	0.085	135 (57.7%)	142 (47.3%)	5.6	0.017
Abuse of antibiotics the main cause of bacterial resistance (Yes)	875 (72.9%)	475 (79.2%)	400 (66.7%)	23.7	0.000	277 (75.7%)	205 (68.3%)	4.5	0.035	198 (84.6%)	195 (65.0%)	20	0.000
Antibiotic resistance affect you and your family’s health	824 (68.7%)	425 (70.8%)	399 (66.5%)	2.6	0.106	247 (67.5%)	188 (62.7%)	1.7	0.194	178 (76.1%)	211 (70.3%)	2.2	0.139
Necessary to get more information about antibiotics	1146 (95.5%)	583 (97.2%)	563 (93.8%)	7.7	0.005	353 (96.4%)	286 (95.3%)	0.53	0.468	230 (98.3%)	277 (92.3%)	9.7	0.002
Need to establish to establish course “Rational use of antibiotics” at the university level	1061 (88.4%)	553 (92.2%)	508 (84.7%)	16.5	0.000	335 (91.5%)	250 (83.3%)	10.4	0.001	218 (93.2%)	258 (86%)	7	0.008

*p < 0.05; #Significance; χ2 = chi square test.; n (%): Frequency (Percentage)

### Practice on antibiotic use: analysis of overall practice score

**Fig. 3:**
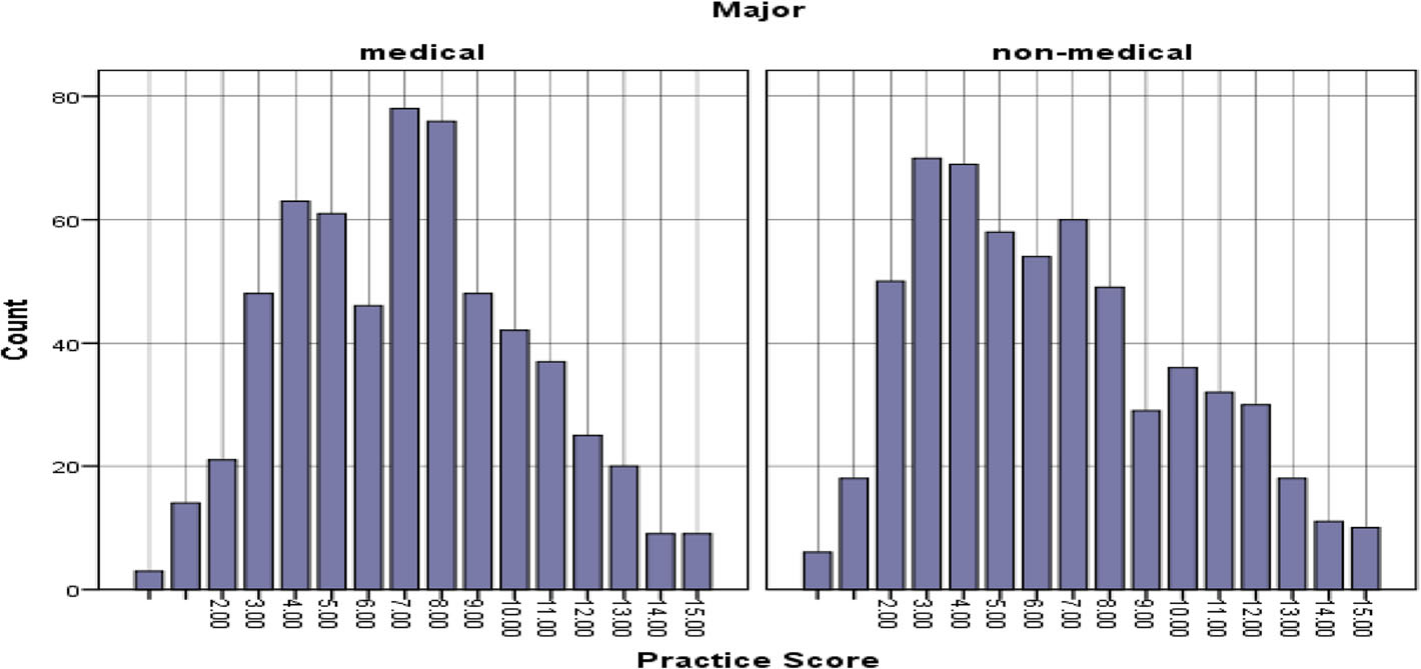
Histogram of practice score by major. Figure 3 shows the distribution of overall practice scores among medical and non medial students. The values in the x axis represents the total number of correct answers. Y axis represents the count

The average practice core was 45% with a 95% CI [44, 47%]. This means that on a practice scale of 0 to 100, participants scored an average of 45 points in the practice of antibiotic use. MS scored relatively higher than NS in the practice of antibiotic use (*p* = 0.002). Their average score was 47% compared to 43% in NS. The same pattern of results was observed between final-year MS and NS (*p* = 0.001) while there was no significant difference between first-year MS and NS (*p* = 0.232) (see Table 1 and Fig. 3). The results of each of the questions related to practice of antibiotic usage among MS and NS students were analysed using χ2 test (see Table 4).

**Table 4:** Number and percentage of the questions on the attitude of antibiotic use

Question (response)	Total % (n/N)	Whole n (%)				1st year n (%)			Final year n (%)				
		MS	NS	χ2	p	MS	NS	χ2	p	MS	NS	χ2	p
Use antibiotics when having fever (temperature lower than 38.5°)	655 (54.6%)	323 (53.8%)	332 (55.3%)	0.27	0.602	210 (57.4%)	175 (58.3%)	0.062	0.804	113 (48.3%)	157 (52.3%)	0.860	0.354
Common cold (always, often	696 (58%)	319 (53.2%)	377 (62.8%)	11.5	0.001	214 (58.5%)	190 (63.3%)	1.6	0.201	105 (44.9%)	187 (62.3%)	16.2	0.000
Acute bronchitis (always, often)	793 (66.1%)	407 (67.8%)	386 (64.3%)	1.6	0.20	231 (63.1%)	189 (63%)	0.001	0.976	176 (75.2%)	197 (65.7%)	5.6	0.017
Pneumonia (always, often)	737 (61.4%)	391 (65.2%)	346 (57.7%)	7	0.008	213 (58.2%)	162 (54)	1.2	0.227	178 (76.1%)	184 (61.3%)	13.1	0.000
Coughing up yellow/green sputum (always, often)	771 (64.3%)	386 (64.3%)	385 (64.2%)	0.004	0.95	229 (62.6%)	189 (63%)	0.013	0.909	157 (67.1%)	196 (65.3%)	0.182	0.670
Sore throat (always, often)	822 (68.5%)	412 (68.7%)	410 (68.3%)	0.015	0.901	260 (71%)	206 (68.7%)	0.441	0.506	152 (65%)	204 (68%)	0.584	0.459
Cough with fever (always, often)	879 (73.3%)	450 (75%)	429 (71.5%)	1.8	0.171	286 (78.1%)	211 (70.3%)	5.3	0.21	164 (70.1%)	218 (72.7%)	0.430	0.512
Congested nose with headache (always, often)	717 (59.8%)	335 (55.8%)	382 (63.7%)	7.6	0.006	224 (61.2%)	190 (63.3%)	0.318	0.573	111 (47.4%)	192 (64%)	14.5	0.000
Coughing up white sputum (always, often)	562 (46.8%)	275 (45.8%)	287 (47.8%)	0.482	0.488	179 (48.9%)	141 (47%)	0.240	0.624	96 (41%)	146 (48.7%)	3.1	0.078
Cough lasting 2 weeks or more (always, often)	804 (67%)	390 (65%)	414 (69%)	2.2	0.141	254 (69.4%)	212 (70.7%)	0.126	0.723	136 (58.1%)	202 (67.3%)	4.8	0.028
Stop the use of antibiotics as soon as complaints lessen (Yes)	534 (44.5%)	208 (34.7%)	326 (54.3%)	47	0.000	152 (41.5%)	156 (52%)	7.3	0.007	136 (58.1%)	202 (67.3%)	57.7	0.000
Antibiotic used prescribed by doctor (No)	433 (36.1%)	185 (30.8%)	248 (41.3%)	14.3	0.000	98 (26.8%)	110 (36.7%)	7.5	0.006	87 (37.2%)	138 (46%)	4.2	0.041
Follow prescription when choose Antibiotic (No)	317 (26.4%)	142 (23.7%)	175 (29.2%)	4.6	0.031	78 (21.3%)	69 (23)	0.273	0. 601	64 (27.4%)	106 (35.3%)	3.8	0.049
Used antibiotics without doctor’s Instructions (Yes)	667 (55.6%)	325 (54.2%)	342 (57%)	0.97	0.323	172 (47%)	160 (53.3%)	2.6	0.104	153 (65.4%)	182 (60.7%)	1.3	0.263
Ask the doctor to prescribe for you antibiotics when you catch a common cold (Yes)	445 (37.1%)	185 (30.8%)	260 (43.3%)	20	0.000	131 (35.8%)	137 (45.7%)	6.7	0.010	54 (23.1%)	123 (41%)	19.1	0.000

*p < 0.05; #Significance; χ2 = chi square test.; n (%): Frequency (Percentage)

## Discussion

As per our knowledge, this is the first large-scale study assessing the KAP of antibiotic use among MS and NS students in the Arab countries, the Gulf region and UAE. On an average, MS and NS scored higher in attitude score followed by knowledge score and practice score. The average attitude score was 76% (95% CI: [75, 78%]) compared to 59% for knowledge score (95% CI: [58, 60%]) and 45% (95% CI: [44, 47%]) for practice score. The study results confirmed statistically significant differences in the KAP of antibiotic use among MS and NS. Regarding knowledge of antibiotic use, the results showed that overall MS scored significantly higher than NS (*p* = 0.000). The same pattern of results was observed between first-year MS and first-year NS (p = 0.000) and final-year MS and final-year NS (p = 0.000). This agrees with other findings about knowledge of antibiotic use reported in the literature [7, 8, 20, 21]. Other studies in Malaysia [9, 13] among final-year dentistry and pharmacy students reported a good knowledge of antibiotic use among medical students. These findings are parallel with the study in Italy [16] amongst students of a school of medicine that reported good knowledge about antibiotics use. Therefore, these results suggest that medical students have more knowledge about antibiotics than other students or the public. However, some studies showed that knowledge of antibiotic use was moderate to poor among medical students [10–12, 22–24].

In this study, although overall MS scored better than NS regarding the knowledge of antibiotic use, MS had poor knowledge that may results in antibiotic abuse. About a third of the MS (34.2%) were confused about whether antibiotics were effective against bacteria or viruses; a similar response was observed in Chinese study [7]. in which 35.5% of respondents were unaware that antibiotics do not cure viral infections, whereas 83.2% of respondents in Italian study [16] and 100% of respondents in Iranian study [12] were conscious that antimicrobial drugs are not appropriate for viral infections. Moreover, two-thirds of the MS (65.8%) believed that antibiotics can speed up the recovery of common cold, cough and a number of other related illnesses arising from viral infections which is relatively high compared to other relevant studies [7, 14]. Another study also found in a telephone survey that 27% of respondents with common cold believed that antibiotics made them recover more quickly whereas in Jamshed et al. [13], only 4.9% believed that common cold and cough should always be treated with antibiotics to make the patient recover faster. This study also demonstrated that nearly half of the MS (47%) perceived that antibiotics are obtainable without interference of a doctor at drug store or at pharmacies. This poor knowledge about antibiotic use reflects the need to promote awareness on this topic during course curriculums.

Regarding the attitude of medical and non-medical students towards antibiotics use, our results showed that overall MS scored higher than NS (*p* = 0.000); this was consistent with other studies on college students [7, 8]. However, a study in Trinidad and Tobago [9] showed a negative attitude of pharmacy students towards antibiotic use and resistance while the [12], majority of medical interns (73.1%) had a moderate attitude towards antibiotic resistance etiology [12],

Our results also showed that first-year MS performed better than first-year NS in their attitude towards antibiotic use (*p* = 0.001) while no significance difference was found between first-year medical and non-medical students in Huang et al. [7]. Moreover, this study showed that final year MS performed better than NS (*p* = 0.000), and this was similar to Huang et al. [7]

Our study showed that 91.3% of medical students believed that thereis an abuse of antibiotics at present; this was consistent with Huang et al. [7] which showed that 90.1% of respondents are aware of this fact. However only 47.8% of our respondents believed that it is a national problem. On the other hand, a study among interns and senior physicians in France showed that 98% of physicians considered antibiotic resistance a national problem [25]. Furthermore, 94% of medical students in Abbo et al. [26] 83% of the Chinese medical students in Huang et al. [7] and 88.65% of Indian medical students in Khan and Banu [14] shared this view. Therefore, it is important to organize educational campaigns to address these issues.

Minen et al. [27] in a KAP survey on antibiotic use among 304 MS in America reported that more than 75% of the students preferred more education on antibiotics which was consistent with 89% of medical students in Huang et al. [7] and 90% of medical students in Abbo et al. [26] These studies support our findings which concludes that 97.2% of MS believed that it is necessary to get more education about antibiotics.

With regard to the practice of antibiotic use, our result showed that overall MS scored significantly higher than NS (*p* = 0.002). The findings are in line with the results presented by the study in Chennai [8] and India [14]. Another study in a school of medicine in Italy [16] concluded that healthcare profession students did not practice what they knew. Moreover, the practice towards antibiotics resistance and prescribing was in moderate range among students [12].

Our study showed also that there were no significance difference between first-year medical and non-medical students regarding the practice of antibiotic usage (*p* = 0.232); this was in accordance with Huang et al. [7] Additionally, this study showed that final year MS performed better than final year NS on practice of antibiotic usage (*p* = 0.001).

An alarming finding was the one related to the overuse of antibiotics for various symptoms of respiratory tract infection among medical students. For instance, more than half of the MS would use antibiotics more frequently for acute bronchitis, pneumonia, coughing up yellow/green sputum, cough, fever, congested nose with headache and cough lasting two weeks or more. This was similar to the study in Nigeria which showed a high rate of consumption of antibiotics among university undergraduates [15]. The study has also revealed that 45.8% of MS showed high frequency of antibiotic usage when coughing up white sputum, and 54.2% of them had used antibiotics without doctor’s instructions. Our study showed that 53.2% of MS used antibiotics for common cold and 68.7% of them used antibiotics for sore throat while in Huang et al. [7] 13.6 and 15.3% of MS used antibiotics for common cold and sore throat, respectively. Moreover, in Khan and Banu [14] 38.1% of respondents agreed to take antibiotics to prevent further serious illnesses when they had common cold. On the other hand, Jorak et al. [12] showed that 100% of the interns knew that antibiotics were not recommended to treat common cold and viral infections, and this was consistent with Scaioli et al. [16] which showed that 99.62% of respondents did not take antibiotics for cold or sore throat as well as with Jamshed et al. [13] which found that 95.1% of the participants aware that common cold and cough should not be treated with antibiotics and Ahmad et al. [9] which concluded that only 8.3% respondents agreed to antibiotics’ help in the prevention of illness during common cold and flu. This implies that the education that medical students rely upon is not sufficient and that better education is required on the appropriate use of antibiotics to improve their practice towards antibiotics and their use.

## Conclusions

Although this study’s findings showed that overall MS performed better than NS on the KAP of antibiotic use, there are some potential causes of antibiotic abuse; this implies that education imparted by our universities on this issue was weak and adding some courses on rational antibiotic use in the medical curriculum is urgently required.

## Abbreviations

GCC: Gulf Cooperation Council
KAP: Knowledge, attitude and practice
UAE: United Arab Emirates
